# Feature tracking cardiac magnetic resonance imaging for the evaluation of myocardial strain in patients with dilated cardiomyopathy and in healthy controls

**DOI:** 10.1186/1532-429X-15-S1-P167

**Published:** 2013-01-30

**Authors:** Kristin Breuninger, Stephanie Lehrke, Philipp Matheis, Yannick Sander, Rebekka Kammerer, Lukas Rust, Christian Galuschky, Hugo A Katus, Grigorios Korosoglou, Sebastian Buss

**Affiliations:** 1 University of Heidelberg, Heidelberg, Germany; 2 Diagnostic and Interventional Radiology and Neuroradiology, Ev.- Lutherische Diakonissenanstalt Flensburg, Flensburg, Germany; 3 TomTec Imaging Systems, Munich, Germany

## Background

In clinical routine, quantification of myocardial strain using CMR tagging is currently the gold standard. Additional pulse sequences for the generation of tagged images and specialized software for the quantification of myocardial strain is necessary, so that alternative ways using conventional steady-state-free-precession (SSFP) sequence images would be preferable. This advantage may be ensured by feature tracking imaging algorithm, a novel method of two-dimensional deformation analysis.

To quantify myocardial deformation with two-dimensional feature tracking cardiac magnetic resonance (CMR) in patients with heart failure due to non-ischemic cardiomyopathy and in healthy controls.

## Methods

Eighty-eight patients with dilated cardiomyopathy and thirty healthy subjects were examined in a 1.5T CMR-scanner. SSFP cine sequences of the four chamber view and mid-ventricular short axis view were analyzed using feature tracking imaging software (2D CPA MR©, TomTec Imaging Systems GmbH). Generated parameters of the myocardial quantification were circumferential and longitudinal strain, respectively. Furthermore, patients were divided in subgroups classified by left-ventricular ejection-fraction LV-EF≤35% and EF>35% and in patients with the presence or absence of late-gadolinium enhancement (LGE), respectively.

## Results

In patients with dilated cardiomyopathy, close correlation were observed for the LV-EF with circumferential strain (r^2=0.8, p<0.0001) and with longitudinal strain (r^2=0.5, p<0.0001), respectively. Regression analysis showed also high correlation between NT-proBNP and circumferential strain (r^2=0.47, p<0.0001). Furthermore, significant differences of circumferential and longitudinal strain parameters were observed between healthy volunteers and patients with LV-EF>35% and with LV-EF ≤35% (p<0.0001). In addition, patients with LGE yielded significant lower circumferential and longitudinal strain values, compared to those without LGE (p<0.0001 and p=0.02, respectively).

## Conclusions

Feature tracking imaging determines global myocardial function in patients with dilated cardiomyopathy and provides further insight into the underlying remodeling processes. Further investigation is necessary to analyze the impact of this new method on clinical outcome.

## Funding

none

